# The single nucleotide variant rs12722489 determines differential estrogen receptor binding and enhancer properties of an *IL2RA* intronic region

**DOI:** 10.1371/journal.pone.0172681

**Published:** 2017-02-24

**Authors:** Marina A. Afanasyeva, Lidia V. Putlyaeva, Denis E. Demin, Ivan V. Kulakovskiy, Ilya E. Vorontsov, Marina V. Fridman, Vsevolod J. Makeev, Dmitry V. Kuprash, Anton M. Schwartz

**Affiliations:** 1 Engelhardt Institute of Molecular Biology, Russian Academy of Sciences, Moscow, Russia; 2 Moscow Institute of Physics and Technology, Moscow, Russia; 3 Vavilov Institute of General Genetics, Russian Academy of Sciences, Moscow, Russia; 4 Skolkovo Institute of Science and Technology, Skolkovo Innovation Center, Moscow, Russia; 5 Faculty of Biology, Lomonosov Moscow State University, Moscow, Russia; University of Texas Rio Grande Valley, UNITED STATES

## Abstract

We studied functional effect of rs12722489 single nucleotide polymorphism located in the first intron of human *IL2RA* gene on transcriptional regulation. This polymorphism is associated with multiple autoimmune conditions (rheumatoid arthritis, multiple sclerosis, Crohn's disease, and ulcerative colitis). Analysis *in silico* suggested significant difference in the affinity of estrogen receptor (ER) binding site between alternative allelic variants, with stronger predicted affinity for the risk (G) allele. Electrophoretic mobility shift assay showed that purified human ERα bound only G variant of a 32-bp genomic sequence containing rs12722489. Chromatin immunoprecipitation demonstrated that endogenous human ERα interacted with rs12722489 genomic region *in vivo* and DNA pull-down assay confirmed differential allelic binding of amplified 189-bp genomic fragments containing rs12722489 with endogenous human ERα. In a luciferase reporter assay, a kilobase-long genomic segment containing G but not A allele of rs12722489 demonstrated enhancer properties in MT-2 cell line, an HTLV-1 transformed human cell line with a regulatory T cell phenotype.

## Introduction

New technologies, modern computational capacities and collection of large study populations through international collaboration brought new hope for understanding the mechanisms of complex diseases with strong genetic component [1]. Genome-wide association studies (GWAS) now include over a million single-nucleotide polymorphisms (SNP) and thousands of participants, allowing identification of SNPs with small effect sizes and putative risk loci in or near genes not previously suspected of being involved in the etiology of a particular disease [2]. However, GWAS alone do not provide any definite answers on disease mechanisms, but deliver candidate SNP data for downstream functional annotation. A strongly associated SNP may not be the causative polymorphism by itself; instead, it could be in a strong linkage disequilibrium with the actual functional SNP or may be linked to the causative copy number variant [2]. Even if the causative variant has been identified, the molecular mechanisms that connect the genetic variant and the disease remain undetermined.

State-of-the-art approach to finding candidate SNPs for functional studies includes successive filtering by several criteria: (i) localization in the vicinity of relevant genes; (ii) mapping to genomic regions with regulatory properties such as DNase I accessibility, evolutionary conservation, appropriate epigenetic signature, ChIP-Seq-confirmed binding of transcription factors; (iii) correlation of SNP haplotype with expression levels of candidate genes. However, such algorithm inevitably filters out many SNPs and may lead to under-recognition of actual causative polymorphisms.

Although the majority of regulatory SNPs are cis-acting [3–5], the localization criterion would not be satisfied by some functional SNPs that belong to long-range cis-regulatory elements and are located more than a million base pairs away from their target genes as in the case of *FTO* locus SNPs and *IRF3* expression [6]. Moreover, expression quantitative trait loci (eQTL) analyses showed that considerable fraction of regulatory SNPs are trans-acting (defined as located more than 5 Mb away from the target gene [3, 4] or on a different chromosome [5]), and at least some of them act in both cis and trans [3, 5]. Nevertheless, the localization criterion is eligible and widely used. A notable fraction of regulatory SNPs identified and confirmed by functional studies belong to proximal cis-regulatory elements and lie within 2 kb up- or downstream of transcription start sites [7].

DNase I accessibility, histone modification patterns and data on transcription factors binding are available only for a limited number of cell types, that do not necessarily include the particular cell type involved in the disease development. Evolutionary conservation data also have limited usability, as many human regulatory elements are absent or differently arranged in other species [8]. Finally, a polymorphism may be functional only in a specific cell type under specific conditions as evident from functional studies of multiple sclerosis (MS)-associated SNPs [9, 10]. In that case, an association of the SNP with its target gene expression level would not be revealed in a typical eQTL study where large cohorts of people are tested and therefore individual analysis of each specific cell type is technically challenging.

A complementary approach is to select candidates for further functional assessment based on *in silico* analysis of a genomic sequence that includes an SNP of interest, which can be especially useful when other filters are marginally applicable. As the majority of disease-associated SNPs are found in non-coding regions [11], their influence on disease predisposition can be mediated through direct or indirect alterations of transcription factor binding to DNA [7]. In particular, sequence analysis predicts how transcription factor binding motifs change upon single-nucleotide substitution [12, 13]. We used this approach to annotate autoimmunity-associated SNPs of *IL2RA* locus. *IL2RA* encodes α subunit of the high affinity IL-2 receptor and is expressed by several populations of lymphocytes. Both enhanced and disrupted IL-2 signaling have been shown to induce autoimmunity in mouse models [14] and its alterations were found in a number of autoimmune diseases in humans [10].

Considering available disease association data, transcription-factor-binding site prediction and epigenetic data, we chose to focus on rs12722489, an SNP from *IL2RA* gene associated with rheumatoid arthritis, multiple sclerosis, Crohn's disease, and ulcerative colitis. Our functional analysis showed that genomic region containing the risk allele of rs12722489 specifically binds human estrogen receptor (ER) α and exerts cell-type-specific enhancer properties.

## Materials and methods

### Computational analysis of transcription factor binding sites

We analyzed preselected SNPs with flanking sequences (25 nucleotides at each side) using PERFECTOS-APE, a specialized software to predict effects of SNPs on transcription factor binding using predefined collection of position weight matrices [15] with the following parameters: HOCOMOCO v10 [16] collection of mono- and dinucleotide position weight matrices (PWMs) (only highest quality matrices, A,B,C and secondary motifs S, were used), 0.0005 as a threshold for motif *P*-value for any of two alternative alleles, and 4 as a threshold for the ratio between the motif *P*-values for two alternative alleles.Triallelic SNPs were virtually split into three twoallelic variants and each pair was analyzed separately.

### Electrophoretic Mobility Shift Assay (EMSA)

The following oligonucleotides were used to create radiolabeled probes:

IL2RA fw 5’-ACTTATCCAAGG(G/A)TCTGAGTGGTCTT-3’,

IL2RA rev 5’-CCCTCCAAGACCACTCAGA(C/T)CCTT-3’;

V1B1 fw 5’-CCTCCAGTCACTGTGACCCAACCACAC-3’,

V1B1 rev 5’-CTGAATGTGTGGTTGGGTCACAGTGACTGG-3’.

The oligos (1.5 pmol/μl each) were annealed by boiling and slow cooling overnight in the buffer containing 0.2 M EDTA, 10 mM Tris-HCl (pH 8.0) and 100 mM NaCl. The annealed oligos were then labeled with α-^32^P dATP at room temperature. Each labeling reaction contained 4.5 pmol oligos, 2 units of Klenow Fragment (Thermo Scientific), Klenow reaction buffer, 3 nmol dCTP+dTTP+dGTP mix and 15 pmol dATP^32^(3uCi/pmol) in a total volume of 15 μl. After 20 min, the reaction was stopped by adding 35 μl of EDTA water solution to the final concentration of 10 mM. The oligos were purified using illustra ProbeQuant G-50 Micro Columns (GE Healthcare Lifesciences).

Each binding reaction contained 10% (v/v) glycerol, 80 mM KCl, 25 mM NaCl, 15 mM Tris (pH 8.0), 0.18 mM EDTA, 1 mM MgCl_2_, 1.1 mM DTT, 50 μM Na_3_VO_4_, 33.3 μg/ml BSA, 150 μg/ml poly-dIdC, 12 μg/ml human recombinant ERα (Thermo Scientific) and1.5 μl of radiolabeled oligonucleotide probe in the total volume of 10 μl. Competitor non-radiolabeled probe, when present, was added to the reaction in 100-fold excess. The mix was incubated for 20 min at room temperature and then loaded to 5% polyacrylamide gel in 0.5xTBE (45 mM Tris base, 45 mM Boric acid, 1 mM EDTA, pH 8.0) pre-run for 1.5 h at +4°C. Electrophoresis was performed at 4°C, 10V/cm. Gels were dried end exposed to phosphor screen overnight. The signal was detected using Phosphorimager Cyclone (Packcard).

### Nuclei and nuclear extracts preparation

The protocol described in [17] was used with some modifications. Briefly, lymphocytes were washed in PBS and the cell pellet was resuspended and incubated for 5 min in 5 volumes of buffer A (0.32 M sucrose, 3 mM CaCl_2_, 2 mM Mg acetate, 0.1 mM EDTA, 10 mM Tris-HCl pH 8.0, 1 mM DTT, 0.5 mM PMSF, 0.5% NP-40, 1x protease inhibitor cocktail (Sigma)) on ice. The pelleted nuclei (14000g, 15 sec, +4°C) were resuspended in the equal volume of buffer B (20 mM HEPES pH 7.9, 25% glycerol, 1.5 mM MgCl_2_, 0.02 M KCl, 0.2 mM EDTA, 0.5 mM DTT, 0.5 mM PMSF, 1x protease inhibitor cocktail) and used for chromatin immunoprecipitation. For pull-down experiments, two volumes of buffer C (20 mM HEPES pH 7.9, 25% glycerol, 1.5 mM MgCl_2_, 0.8 M KCl, 0.2 mM EDTA, 1% NP-40, 0.5 mM DTT, 0.5 mM PMSF, 1x protease inhibitor cocktail) was then added to the nuclei, followed by incubation on ice for 10 min. Extracts cleared by centrifugation (14000g, 20 min, +4°C) were snap-frozen and stored in liquid nitrogen.

### DNA pull-down assay

DNA pull-down was performed essentially as described in [18]. DNA probes for pull-down assay were produced by PCR using the following primers: forward 5’-AGGAAGGTTTTTCAATGTGATTTCTACATC and reverse 5’-TTCCCCTGCTCCCTCCAAGAC for putative ERE from *IL2RA* containing rs12722489 (product length 189bp); forward 5’-GTTCTTTTTGTTCTAAATCAGGCTGTA and reverse 5’- TTGGGGATGACACCCGGCTG for internal control (product length 176bp). DNA fragments containing putative ERE from *IL2RA* were amplified using 1 kb (G) and (A) luciferase reporter constructs as a template. Control probes were amplified using a plasmid bearing another 1 kb segment of *IL2RA* first intron as the enhancer and contained ETS-family transcription factor binding site. The resulting PCR products were run in 1.5% agarose gels and purified with GeneJET Gel Extraction Kit (Thermo Scientific). 100 ng of purified rs12722489 oligonucleotides, 100 ng of internal control oligonucleotides, and 10 μl of MT-2 nuclear extract (50 μg total protein) were dissolved in incubation buffer (60 mM KCl, 12 mM HEPES pH 8.0, 4 mM Tris HCl pH 8.0, 0.5 mM EDTA, 5% (v/v) Glycerol) containing 5 μg of sonicated salmon sperm DNA (ssDNA), 1x protease inhibitor cocktail (Sigma) and 1 mM DTT. After 1 hour on ice, incubation conditions were changed to those of RIPA buffer. 2 μg of anti-ERα mouse monoclonal IgG1 antibodies (Abcam, clone C-542) or the same amount of mouse IgG1 isotype control (Abcam) was then added and the probes were incubated for 1 hour on ice. After that, 60 μl of Protein A Mag sepharose (GE Healthcare) beads (washed with RIPA buffer and pre-absorbed with 75 ng/μl beads ssDNA and 0.1 μg/μl beads BSA according to Abcam X-ChIP protocol) was added to each sample and incubation upon rotation was carried out overnight at +4°C. The next day the complexes were washed twice with buffer 1 (0.1% SDS, 1% Triton X-100, 2 mM EDTA, 20 mM Tris pH 8.0, 150 mM NaCl), once with buffer 2 (same as buffer 1 but with 500 mM NaCl) and once with Tris-EDTA (10 mM Tris, 1 mM EDTA, pH 8.0). DNA was then eluted with 20 μl of 2.5% acetic acid and neutralized with 7 μl of 10% sodium bicarbonate. Eluted DNA was analyzed by real-time PCR using qPCRmix-HS SYBR+LowROX kit (Evrogen, Moscow, Russia) and Applied Biosystems^®^ 7500 Real-Time PCR System (Thermo Scientific). The primers were the same as for synthesis of the oligonucleotides. Relative rs12722489 DNA amount was normalized to internal control.

### Chromatin Immunoprecipitation (ChIP)

ChIP was performed as described in [18] with some modifications. Briefly, 5×10^6^ Jurkat or MT-2 cells were treated with 1.2% formaldehyde in complete RPMI medium for 10 minutes at +37°C. The reaction was stopped by adding glycine to the final concentration of 125 mM. Nuclei were isolated as described above and diluted in 130 μl of TE buffer. Sonication of chromatin was performed in accordance with the manufacturer's recommendations using a S220 Focused-ultrasonicator (Woburn, USA) to obtain 500 bp DNA fragments. Sonicates were clarified by centrifugation and stored at -80°C. 5 μl of Protein A Mag sepharose (GE Healthcare) prewashed with TBS was incubated with 3 μg of anti-ERα mouse monoclonal IgG1 antibodies (Abcam, clone C-542) or the same amount of mouse IgG1 isotype control (Abcam) in 300 μl of TBS at +4°C overnight upon rotation, followed by incubation with 20 μl of a sheared chromatin sample for 1–2 h at +4°C upon rotation. Complexes were washed twice with buffer 1 (0.1% SDS, 1% Triton X-100, 2 mM EDTA, 20 mM Tris pH 8.0, 150 mM NaCl), once with of buffer 2 (same as buffer 1 but with 500 mM NaCl) and once with Tris-EDTA (10 mM Tris, 1 mM EDTA, pH 8.0). Elution was performed with 50 μl of 2.5% acetic acid and neutralized with 17.5 μl of 10% sodium bicarbonate. Immunoprecipitated DNA or input chromatin (2 μl) was diluted in TE, digested with 200 μg/ml RNase A for 1 h at 55°C, and treated with 200 μg/ml proteinase K and 0.5% SDS for 2 h at 55°C. Cross-links were reversed by shaking overnight at 65°C. DNA was extracted with phenol/1-bromo-3-chloropropane (Sigma) and diluted in 20 μl of TE buffer. Immunoprecipitated DNA was analyzed by real-time PCR (the same primers as for pull-down assay) using qPCRmix-HS SYBR+LowROX kit (Evrogen, Moscow, Russia) and Applied Biosystems^®^ 7500 Real-Time PCR System (Thermo Scientific). Relative DNA quantity was normalized to input DNA.

### Cells

Jurkat cell line and HTLV-1 transformed cell line MT-2 were obtained through the NIH AIDS Research and Reference Reagent Program. The authenticity of Jurkat and MT-2 cells was confirmed by STR DNA profiling (GORDIZ, Moscow, Russia). Both cell lines have G/G homozygous genotype at rs12722489 as assessed by Sanger sequencing. Cells were cultured in RPMI 1640 medium (Paneco, Moscow, Russia) supplemented with 10% FBS (Biological Industries, Kibbutz, Israel), 2 mM L-glutamine (HyClone), MEM Non-Essential Amino Acids Solution (HyClone), 10 mM HEPES (HyClone), 100 U/ml Penicillin, 100 μg/ml Streptomycin (Gibco) at 37°C and 5% CO_2_.

### Luciferase reporter constructs

Human *IL2RA* promoter (chr10:6,104,115–6,104,700), 1 kb intronic region containing rs12722489 (chr10:6,101,364–6,102,272) and a control region from STAT3 gene (chr17:40508494–40509570; all genomic coordinates are given for GRCh37/hg19 assembly) were amplified by PCR using genomic DNA from Jurkat cells as a template and specific primers containing the indicated cloning sites:

Promoter:

5’-ATATAAGCTTGCTGCCTGACCAGAATCTTG (HindIII),

5’-ATCCATGGCTTCCTGACCCTTGGGAC (NcoI)

Intronic region:

5’-AAGGATCCGCTGTACCCAGTGCGTAG (BamHI),

5’-TATGTCGACTACTGCAAAGTGGCTATGAAG (SalI)

Control:

5’-AGGATCCGGATTACAGGTGTATTTCACCAT (BamHI),

5’-TATGTCGACGTTGATGTAATTCCTTTAAATCTAT (SalI).

The A variant of rs12722489 was introduced into the 1 kb intronic fragment by overlap extension PCR using the following mutation-introducing primers: 5’-AAGGATCTGAGTGGTCTTGGAGG and 5’-CACTCAGATCCTTGGATAAGTCAC.

The *IL2RA* promoter was cloned into pGL3-basic luciferase reporter construct (Promega) using HindIII/NcoI restriction sites. Then 1 kb putative enhancer sequences were cloned immediately downstream of the luciferase gene using restriction sites indicated above. All constructs were verified by Sanger sequencing.

Control ERE-LUC reporter plasmid was kindly provided by Dr. George Reid and Dr. Frank Gannon [19].

### Transfection and luciferase reporter assay

Cells were transfected using Neon^®^ Transfection System (Invitrogen). Two million cells were resuspended in 100 μl of electroporation buffer R that contained 0.5μg of pRL-CMV Renilla luciferase control reporter vector (Promega) and 5 μg of a pGL3-based test vector or control ERE-LUC vector. The procedure was conducted according to the manufacturer’s protocol with electroporation options recommended for each cell line (three 10 ms, 1350 mV pulses for Jurkat; one 30 ms, 1400 mV pulse for MT-2 cells). Luciferase activity was measured 24 h after transfection using Dual-Luciferase Reporter Assay System (Promega) according to the manufacturer’s protocol. Luminescence was detected at Luminometer 20/20n (TurnerBioSystems, USA).

### Estrogen induction

After electroporation, cell probes were divided into five equal aliquots seeded into separate wells of a 12-well culture plate containing estrogen-depleted medium (RPMI 1640 without phenol red supplemented with 10% charcoal-stripped FBS; other components as described above). The next day, 17beta-estradiol (E2) (Sigma) was added to the final concentrations of 10^-9^M, 10^-8^M, 10^-7^M, and 10^-6^M. Luciferase activity was measured 18 h later. Relative Fluc/Rluc signal was normalized to the appropriate untreated control.

### Statistical analysis

To compare luciferase activity levels between different reporter constructs one-sided t-test for independent samples was used. To calculate significance of estrogen influence on luciferase expression one-sided t-test for paired samples was used. The difference was considered significant at *P*-value < 0.05.

## Results

As a case study, we considered *IL2RA* locus, which was one of the first to emerge from early GWAS as an autoimmunity risk factor [20]. We extracted SNP-disease association data from the Genome-Wide Repository of Associations between SNPs and phenotypes (GRASP) [21] and filtered SNPs significantly associated (P < 0.001) with more than one autoimmune disease. The resulting list contained 6 SNPs (Table 1).

**Table 1:** SNPs of the *IL2RA* locus significantly associated with two or more autoimmune diseases according to GRASP database [21].

SNP ID	Location	In LD (r^2^≥0.2) with*:	Minor allele frequency	Risk allele	Phenotype (best *P*-value)
rs3134883	Intron 1	rs3118470 (r^2^ = 0.89), rs706779 (r^2^ = 0.41), rs706778 (r^2^ = 0.62)	A; 0.2143	A	Rheumatoid arthritis (8.6×10^−6^), Alopecia areata (1.1×10^−12^), Primary sclerosing cholangitis (7.3×10^−7^), Vitiligo (1.0×10^−5^)
rs3118470	Intron 1	rs3134883 (r^2^ = 0.89), rs706779 (r^2^ = 0.45), rs706778 (r^2^ = 0.68)	C; 0.3182	C	Type 1 diabetes (1.3×10^−6^), Rheumatoid arthritis (9.2×10^−7^), Alopecia areata (1.7×10^−12^), Multiple sclerosis (3.2×10^−11^)
rs12722489	Intron 1	rs2104286 (r^2^ = 0.56)	T; 0.0962	C	Rheumatoid arthritis (5.3×10^−4^), Multiple sclerosis (3.0×10^−8^), Crohn's disease (2.9×10^−9^), Ulcerative colitis (8.9×10^−4^)
rs2104286	Intron 1	rs12722489 (r^2^ = 0.56)	C; 0.1378	T	Multiple sclerosis (3.5×10^−10^), Rheumatoid arthritis (1.0×10^−3^), Primary sclerosing cholangitis (8.7×10^−4^)
rs706779	Intron 1	rs3134883 (r^2^ = 0.41), rs3118470 (r^2^ = 0.45), rs706778 (r^2^ = 0.43)	C; 0.4721	T	Type 1 diabetes (9.3×10^−8^), Alopecia areata (1.3×10^−3^), Graves' disease (2.3×10^−6^)
rs706778	Intron 1	rs3134883 (r^2^ = 0.62), rs3118470 (r^2^ = 0.68), rs706779 (r^2^ = 0.43)	T; 0.4849	T	Rheumatoid arthritis (1.4×10^−11^), Alopecia areata (4.9×10^−10^), Primary sclerosing cholangitis (5.0×10^−6^)

LD, linkage disequilibrium.

* r^2^ values are provided according to HaploReg v.4.1 [22].

For these SNPs we performed computational analysis of transcription factor binding sites that might be significantly affected by alternating allelic variants. We employed PERFECTOS-APE software [15] to assess motif *P*-value changes for alternative alleles using HOCOMOCO v10 [16] collection of mono- and dinucleotide PWMs. The overall annotation of putative binding sites that have significantly different predicted affinity for alternative allelic variants is provided in S1 Table.

The collection of dinucleotide PWMs is notably smaller, however it provides more accurate predictions as dinucleotide PWMs account for dependencies between neighboring nucleotides [23]. We focused on the only two SNPs that had consistent predictions across mono- and dinucleotide PWMs. The binding sites of ERα and β and Vitamin D3 receptor (all belonging to the nuclear receptor family of transcription factors) showed significant differences in predicted affinity for the two alternative alleles of rs12722489. The AP2A binding site was predicted to be significantly altered by rs706779. Of interest, both SNPs lie inside the T cell-specific super-enhancer [24].

Next, we considered epigenetic data obtained by the Roadmap Epigenomics Consortium [25], specifically histone 3 lysine 4 monomethylation signal (H3K4me1) associated with enhancer regions, and histone 3 lysine 27 acetylation signal (H3K27ac) associated with increased activation of enhancer and promoter regions displayed for the 10 kb area centered on each SNP. The data is summarized in Table 2. The polymorphism rs12722489 is co-located with H3K4me1 enhancer mark only in leukocytes, but not other cell types, and it is also co-located with increased activation mark H3K27ac in memory T cells. Conversely, enhancer marks at rs706779 are less immune-cell specific and are absent in T cells with low *IL-2RA* expression. Therefore we chose rs12722489 for further experimental evaluation.

**Table 2:** Epigenetic marks located at rs12722489 and rs706779 according to the Roadmap Epigenomics Consortium data [25].

	Sample	H3K4me1		H3K27ac	
		rs12722489	rs706779	rs12722489	rs706779
T cells with high *IL2RA* expression	CD4+ CD25- IL17+ PMA-Ionomycin stimulated Th17	+	++	+++	++
	CD4+ CD25+ CD127- Treg Primary Cells	+	+	++	++
Other T cells	CD4+ CD25- CD45RA+ Naive Primary Cells	+			
	CD4+ Naive Primary Cells	+			
	CD4+ CD25int CD127+ Tmem Primary Cells	+		+	
	CD4+ Memory Primary cells	+		+	
	CD4+ CD25- CD45RO+ Memory Primary Cells	+		+	
	CD8+ Naive Primary Cells				
	CD8+ Memory Primary Cells				
Other leukocytes	CD19+ Primary Cells	++	+++		
	CD56+ Primary Cells	+	+		+
	CD14+ Primary Cells				
Other tissues*	Neurospheres, Ganglionic Eminence Derived		+		
	Fetal Adrenal Gland		+		

Histone 3 lysine 4 monomethylation (H3K4me1) is associated with enhancer regions, histone 3 lysine 27 acetylation (H3K27ac) is associated with increased activation of enhancer and promoter regions [25].

(+), (++), (+++) designate relative peak height in arbitrary units.

**Fig 1:**
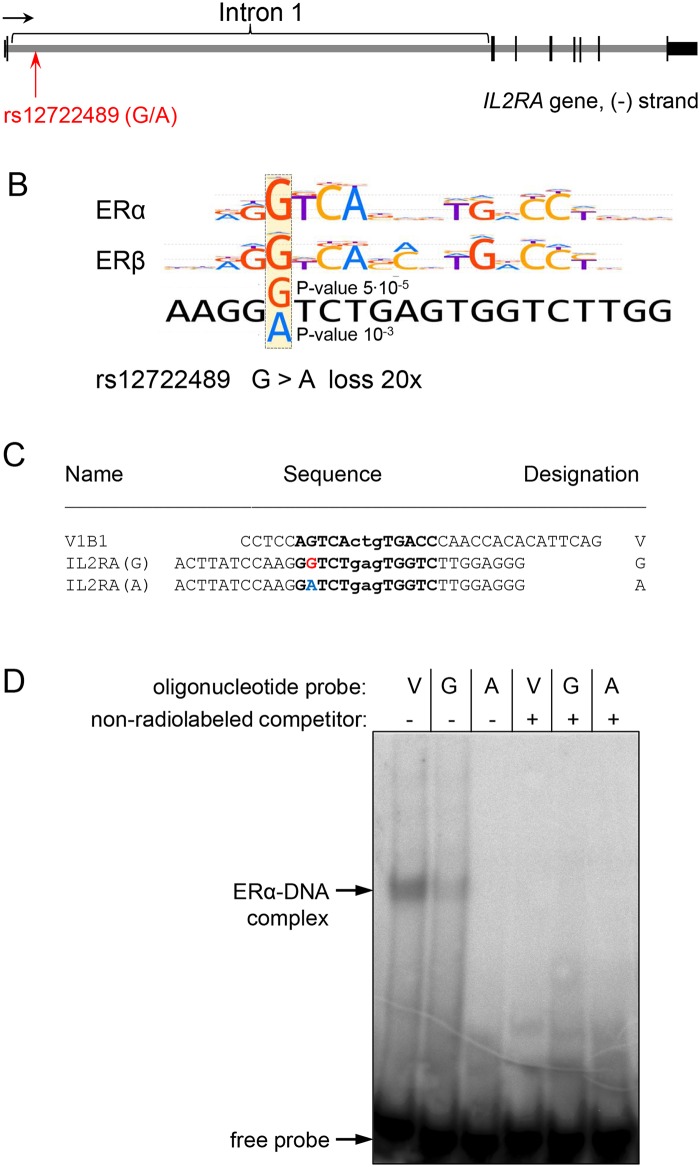
Purified human ERα selectively binds to the genomic sequence containing G variant of rs12722489. (A) Position of rs12722489 in *IL2RA* gene. (B) ERα and β binding motifs displayed as motif logos from HOCOMOCO v10 [16]. Aligned sequence from *IL2RA* gene containing putative ER binding site around rs12722489 is shown underneath. Motif P-values indicated for alternative alleles refer to ERα. The major (risk) allele is shown in red, the minor (protective) allele—in blue. (C) Sequence of DNA probes used for electrophoretic mobility shift assay (EMSA). Genomic sequences are given by the chromosome (-) strand. ER-binding sites are shown in **bold**. The variable nucleotide is shown in color. (D) EMSA was performed using human recombinant ERα and indicated radiolabeled probes. ERα-DNA complex can only be seen for the control V1B1 oligonucleotide containing estrogen-response element from *Xenopus laevis* vitellogenin gene B1 5' flanking region and for the 32 bp genomic sequence containing G variant of rs12722489.

We applied EMSA to compare the capacity of G and A variants of rs12722489 in the context of 32 bp genomic sequence (Fig 1) to bind purified human ERα. This transcription factor has the highest motif *P*-value and its affinity is predicted to be the most altered by G to A single-nucleotide substitution. A well-characterized estrogen response element (ERE) from Xenopus laevis vitellogenin gene B1 5' flanking region [26] was used as a positive control. ERα formed complexes with the 32 bp sequence from human *IL2RA* gene containing the G variant of rs12722489, though the binding was weaker than binding with a canonical ERE (Fig 1D). This appears to be an exception to the rule derived by Driscoll *et al*. from their experimental data which suggested that EREs with three or more changes from the core 13-bp consensus 5’-GGTCAnnnTGACC do not bind ER regardless of the flanking regions context [26]. Binding of the protective A variant of rs12722489 was below detection limit in agreement with the *in silico* prediction.

**Fig 2:**
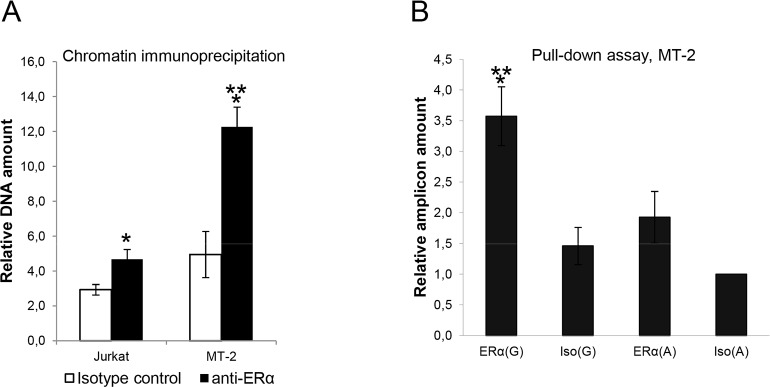
Endogenous ERα binds genomic region containing rs12722489 and binding efficiency depends on the allelic variant. (A) Chromatin immunoprecipitation was performed in Jurkat and MT-2 cells using antibodies to human ERα. Precipitated DNA was analyzed by real-time PCR using primers specific to a 189-bp genomic sequence containing rs12722489. *p<0.*05* comparing to isotype control. **p<0.*05* comparing to Jurkat cells. (B) DNA pull-down assay was performed using MT-2 nuclear extract, 189-bp amplicons from human *IL2RA* gene containing rs12722489 allelic variants, and antibodies to human ERα. *p<0.*05* comparing to isotype control. **p<0.*05* comparing to the G variant. Data from at least 3 independent experiments are represented as mean±SEM.

To test if endogenous ERα binds *IL2RA* intronic region containing rs12722489 *in vivo*, we performed ChIP experiments with nuclei of Jurkat and MT-2 cells. We chose these two cell lines because 1 kb genomic sequence containing rs12722489 has been shown to exert allele-dependent enhancer properties only in MT-2 but not in Jurkat cells (see below). In both cell lines endogenous ERα bound DNA around rs12722489. In MT-2 cells anti-ERα precipitated about 2.5 times more corresponding DNA than isotype control, whereas in Jurkat cells this factor was only 1.5 (Fig 2A).

The differential binding of human ERα to alternative rs12722489 alleles was confirmed by DNA pull-down assay using nuclear extracts from MT-2 cells, antibodies to human ERα and amplified 189-bp genomic sequence embedding rs12722489. Binding of the A variant was near background level, whereas G variant bound two times more efficiently (Fig 2B).

**Fig 3:**
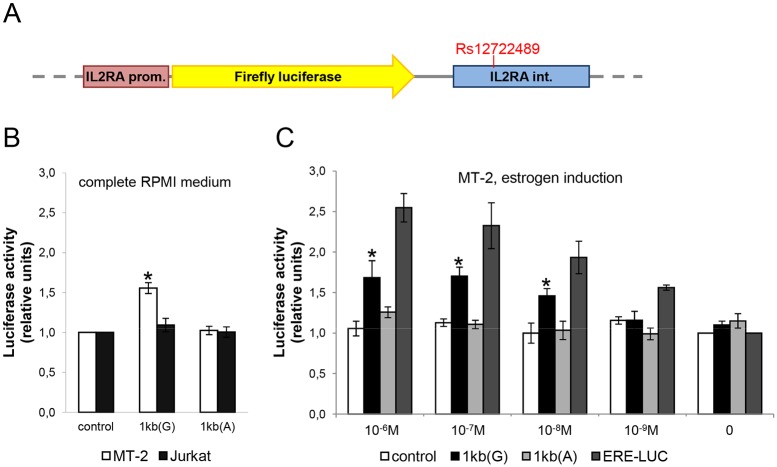
Enhancer activity of the 1 kb *IL2RA* intronic region containing rs12722489 depends on the single nucleotide variant and is estrogen-dependent. (A) Design of the pGL3-based vectors used for luciferase reporter assay. The constructs contained either G or A variant of the 1kb fragment of *IL2RA* first intron downstream of the luciferase gene. The control vector (not shown on the figure) contained an irrelevant 1 kb sequence (see Materials and methods section for details). Position of rs12722489 within the 1kb intronic region is indicated. (B) MT-2 or Jurkat cells grown in complete RPMI medium were transiently transfected with the luciferase reporter constructs. *p<0.*05* comparing to control or 1kb(A) construct. (C) MT-2 cells transfected with the constructs indicated in the legend were placed in steroid-free medium and 17β-estradiol (E2) was added 24h later at the specified concentrations. Luciferase signal was assessed 18h after E2 addition. *p<0.*05* comparing to cells without E2. Data from at least 3 independent experiments are represented as mean±SEM.

To test the effect of the single nucleotide variation on initiation of transcription, we cloned a 1 kb region of *IL2RA* first intron containing rs12722489 to a pGL3-derived vector containing firefly luciferase gene under *IL2RA* promoter (Fig 3A). As increase in plasmid size dramatically affects the luciferase signal [27], our control vector contained irrelevant 1 kb sequence from *STAT3* gene with no enhancer properties. The *IL2RA* promoter (-367 to +218 nucleotides from the major transcription start site) was chosen as an evolutionary conserved genomic region having appropriate epigenetic and DNase hypersensitivity signature in lymphoid cell lines according to the ENCODE data (S1 Fig) [28] visualized in UCSC Genome Browser (http://genome.ucsc.edu, [29]). It includes positive regulatory region I (-276 to -244) which contributes to the inducibility of the *IL2RA* gene, and positive regulatory region II (-137 to -64) involved in basal promoter activity as well as in T-cell-specific expression [30].

We tested our reporter constructs in two cell lines, Jurkat and MT-2. Jurkat is a T cell leukemia-derived cell line that retains its original T-helper properties with regard to surface antigen expression and inducibility of the IL-2/IL-2Rα positive feedback loop [31]. MT-2 is a human T-cell leukemia virus type 1 (HTLV-1)-infected cell line that has the phenotypic and functional characteristics of human regulatory T cells (Tregs) and as such demonstrate high levels of constitutive *IL2RA* expression [32].

The G variant of the 1 kb sequence enhanced luciferase expression in MT-2 cells grown in complete RPMI medium by about 50%, whereas G to A substitution abrogated this effect. In Jurkat cells, the 1kb fragment failed to demonstrate enhancer activity (Fig 3B). We performed estrogen induction experiments to confirm that the observed effect was estrogen receptor dependent. Cells were placed in steroid-free medium immediately after transfection, and 17β-estradiol (E2) at concentrations from 10^−9^ to 10^−6^ M was added 24h later. Luciferase signal was assessed 18h after E2 addition. As a control for adequate estrogen induction and the presence of functional ER in MT-2 cells, part of the cells in each experiment were transfected with a highly estrogen-responsive control ERE-LUC vector which gave dose-dependent induction. In this experimental system, 1 kb (G) did not demonstrate enhancer properties in the absence of E2 whereas addition of estrogen at 10^−9^–10^-6^M resulted in up to 1.5-fold increase in luciferase signal. The A variant of the 1 kb sequence had no effect on luciferase expression regardless of the E2 presence in culture medium (Fig 3C).

## Discussion

International Multiple Sclerosis Genetics Consortium initially reported rs12722489 as the strongest MS-associated SNP in the *IL2RA* locus in 2007 [33]. Yet, the study on a larger data set indicated that this association was secondary to that of a nearby rs2104286 which is in moderate linkage disequilibrium with rs12722489 (r^2^ = 0.56) [34]. Later studies gave conflicting results as several reports confirmed the association of rs12722489/rs2104286 with MS onset [35–38], while others revealed no such association [39, 40], and one report demonstrated correlation with MS progression only [41]. Variants at rs12722489 and rs2104286 affected soluble IL-2Rα concentration in healthy controls but not in MS patients [42] although no correlation with mRNA level was observed [43].

Concurrently, strong association of rs12722489 with Crohn's disease was reported [44]. Interestingly, rs2104286 did not emerge as associated with this disease at all. In type 1 diabetes (T1D), no association with rs12722489 has been reported but rs2104286 has been shown as associated with T1D [42].

Thus, different GWASs give confusing information on the significance of a given polymorphism and this makes complementary analytical tools for identification of candidate SNPs (such as transcription factor binding site prediction) even more important.

Analysis using PERFECTOS-APE software showed that rs12722489 was located in a very conservative position of a putative ER binding site (Fig 1B) and G to A substitution was predicted to destroy the motif (S1 Table). This position of ER binding site is involved in sequence-specific interaction of the ER zinc finger CI with the DNA major groove [45]. Our prediction is biologically credible as ERα, β1 and β2 are indeed present in human leukocytes including peripheral T and B cells [46–48] and have prominent effects on immune function [49]. Of interest, human leukocytes contain not only full-length mRNAs of these two ER isoforms but also a number of exon-deleted splice variants of ERα and β which are supposed to have distinct functions [48, 50].

The *IL2RA* gene encodes CD25 protein which represents α subunit of the high-affinity IL-2 receptor. The high affinity IL-2 receptor is best known for its role in T cell development and function. CD25 is constitutively expressed at high levels by CD4+ Foxp3+ Tregs and is induced on naïve and memory T cells upon antigen stimulation [51]. It can be detected on some memory T-cells at low levels even in the absence of activation [9, 14]. Other immune cells also express CD25 on their surface as was shown for dendritic cells [52–54], activated B cells [55] and activated monocytes [56, 57]. Both enhanced and disrupted IL-2 signaling can induce autoimmunity, indicating that there is a necessity for the maintenance of an optimal level of IL-2/IL-2R signaling given the multiple functions of this pathway [14]. Lymphoid tissue inducer cells (LTi cells) is another interesting subpopulation that express CD25 and was found in elevated numbers in the cerebrospinal fluid of MS patients [58–60].

Since changes in *IL2RA* expression can have opposing outcomes in terms of disease susceptibility depending on the cell type, it is of great importance to reveal in which cell type a particular causative SNP plays its role. This would also be crucially important if gene therapy of autoimmune disorders ever reaches practical use in clinic.

Using luciferase reporter system we showed that the 1 kb genomic segment containing rs12722489 increased *IL2RA* promoter activity in MT-2 but not in Jurkat cells and that this SNP affected luciferase expression only in the former cell type. This is in agreement with the observation that the majority of cell type-specific effects of common genetic variation on gene expression results from cell type-specific use of regulatory elements [61]. DNA region containing rs12722489 is less occupied by ERα in Jurkat cells than in MT-2 (Fig 2A) and experiments with the control ERE-LUC vector showed that Jurkat cells had low potency to respond to estrogens (S2 Fig).Therefore, inability to demonstrate enhancer properties by the 1kb sequence containing rs12722489 in Jurkat cell line could be at least partially explained by the low activity of ERα in these cells, although contribution of other factors cannot be excluded.

## Supporting information

S1 Table Predicted binding sites that have significantly different affinity for alternative allelic variants of selected SNPs (PERFECTOS-APE predictions with HOCOMOCO v10). The predictions overlapping between mono- and dinucleotide PWMs are marked in green
(XLSX)

S1 Fig A view of *IL2RA* promoter region in the UCSC Genome Browser (http://genome.ucsc.edu), hg38 assembly. The promoter region indicated by a blue bar and the tracks considered in promoter region selection shown below.
(TIF)

S2 Fig Jurkat cells have low potency to respond to estrogen. Jurkat cells were placed in steroid-free medium immediately after transfection with ERE-LUC plasmid and 17β-estradiol (E2) was added 24h later. Luciferase signal was assessed 18h after E2 addition. Data is represented as mean±SD.
(TIF)
