# Supplementary figures and images for: The single nucleotide variant rs12722489 determines differential estrogen receptor binding and enhancer properties of an IL2RA intronic region
Source: PLoS One. 2017 Feb 24;12(2):e0172681. doi: 10.1371/journal.pone.0172681 (PMC5325477; doi:10.1371/journal.pone.0172681)

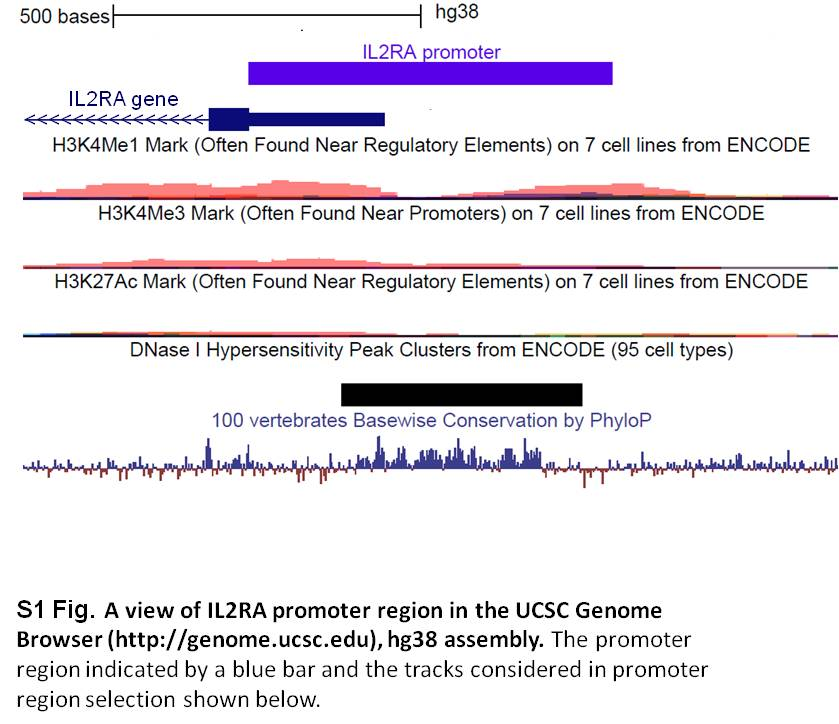

Supplement: S1 Fig — The promoter region indicated by a blue bar and the tracks considered in promoter region selection shown below. (TIF) [file pone.0172681.s002.tif]
